# The spike gene target failure (SGTF) genomic signature is highly accurate for the identification of Alpha and Omicron SARS-CoV-2 variants

**DOI:** 10.1038/s41598-022-21564-y

**Published:** 2022-11-08

**Authors:** Tracy McMillen, Krupa Jani, Elizabeth V. Robilotti, Mini Kamboj, N. Esther Babady

**Affiliations:** 1 Department of Pathology and Laboratory Medicine, Memorial Sloan Kettering Cancer Center, 327 East 64th Street, New York, NY 10065 USA; 2 Department of Medicine, Memorial Sloan Kettering Cancer Center, 327 East 64th Street CLM 522, New York, NY 10065 USA

**Keywords:** Clinical microbiology, Infectious-disease diagnostics

## Abstract

The Alpha (B.1.1.7) and Omicron (B.1.1.529, BA.1, BA.4 and BA.5) variants of concern (VOC) share several mutations in their spike gene, including mutations resulting in the deletion of two amino acids at position 69 and 70 (del 69–70) in the Spike protein. Del 69–70 causes failure to detect the S gene target on a widely used, commercial test, the TaqPath SARS-CoV-2 RT-PCR (Thermo Fisher). The S gene target failure (SGTF) signature has been used to preliminarily infer the presence of Alpha and Omicron VOC. We evaluated the accuracy of the SGTF signature in identifying these two variants through analysis of all positive SARS-CoV-2 samples tested on the TaqPath RT-PCR and sequenced by next generation sequencing between December 2020 to July 2022. 2324 samples were successfully sequenced including 914 SGTF positive samples. The sensitivity and specificity of the SGTF signature was 99.6% (95% CI 96.1–99.9%) and 98.6% (95% CI 99.2–99.8%) for the Alpha variant and 99.6% (95% CI 98.9–99.9%) and 99.8% (95% CI 99.4–99.9%) for the Omicron variant. At the peak of their corresponding wave, the positive predictive value of the SGTF was 98% for Alpha and 100% for Omicron. The accuracy of the SGTF signature was high, making this genomic signature a rapid and accurate proxy for identification of these variants in real-world laboratory settings.

## Introduction

Since its emergence in December 2019, the Severe Acute Respiratory Syndrome Virus 2 (SARS-CoV-2) has caused over 550,000,000 infections and over 6,000,000 deaths worldwide^1^. As SARS-CoV-2 evolved, the World Health Organization (WHO) and the United States Centers for Disease Control and Prevention (CDC) developed criteria to classify SARS-CoV-2 variants based on their potential for increased transmissibility, virulence, disease severity and decreased response to public health measures such as diagnostic tests and vaccines. Variants are classified as variant of interest (VOI), variant of concern (VOC) or variant under monitoring (VUM)^2,3^.

The first SARS-CoV-2 VOC was identified in the United Kingdom (UK) in the Fall of 2020^4^. The initial clue on the emergence of this SARS-CoV-2 variant was the failure of a commonly used commercial test, the TaqPath COVID-19 combo test (Thermo Fisher Scientific, Waltham, MA), to detect the SARS-CoV-2 spike gene (S gene) target sequence in samples positive for SARS-CoV-2 RNA. The TapPath COVID-19 combo test is a real-time reverse transcriptase (RT), multiplexed PCR (RT-PCR) designed to detect sequences of three SARS-CoV-2 genes including the S gene, the open reading frame (ORF) gene and the Nucleocapsid (N) gene. A sample tested on the TaqPath RT-PCR is resulted as positive if at least 2 of the 3 genes target sequences are detected. In October 2020, the UK Health Agency reported increased detection of samples with positive for both ORF and N genes but not the S gene. This S gene target failure (SGTF) quickly became a proxy for the presence of the emerging VOC^4^. Whole genome sequencing of SGTF samples revealed the presence of several mutations in this novel variant, including nucleotides deletions in the S gene resulting in deletion of amino acids at position 69 and 70 (del 69–70) in the Spike protein. These deletions caused the SGTF signature observed on the TaqPath RT-PCR test. The VOC was assigned the B.1.1.7 Pango lineage and designated Alpha using the WHO Greek letter guidance for naming VOC^3^. As the worldwide prevalence of the Alpha variant increased, detection of the SGTF by the TaqPath RT-PCR or other similar tests was used as a proxy for its presence^5^. By May 2021, a new VOC, B.1.617.2 or Delta, emerged, replacing Alpha and becoming the dominant VOC by the Fall of 2021^3^. Since Delta did not have the same del 69–70 in its S gene, samples positive with Delta could be differentiated from those positive for Alpha based on the presence or absence of the SGTF signature.

On November 26, 2021, the WHO classified Omicron as a VOC^6^. Omicron was first identified by the South African lancet laboratories in Pretoria and currently includes the Pango lineage B.1.1.529 with additional notable subvariants including BA.1, BA.1.1, BA.2, BA.3, BA.4 and BA.5^7^. Similar to the Alpha variant, several Omicron subvariants (B.1.1.529, BA.1, BA.4 and BA.5) are characterized by the presence of the SGTF signature due to the spike gene del 69–70^8^.The emergence and disappearance over time of different VOC, with or without the SGTF signature, has reinforced the utility of this PCR result pattern for the initial and rapid classification of VOC. However, systematic studies analyzing the accuracy of the SGTF signature are sparce.

In this study, we investigated the accuracy of the SGTF genomic signature for the detection of the Alpha and Omicron variants (B.1.1.529, BA.1, BA.4 and BA.5) at a tertiary care cancer center in New York City.

## Methods

### Setting and study samples

Memorial Sloan Kettering Cancer Center (MSKCC) is a 514-bed tertiary care cancer center in New York City. SARS-CoV-2 testing using a laboratory-developed test (LDT), became available at MSKCC in March 2020^9^. In October 2020, the LDT was replaced with the TaqPath COVID-19 Combo kit (Thermo Fisher Scientific, Waltham, MA) and used, along with the Cobas SARS-CoV-2 test (Roche Molecular Diagnostics, Indianapolis, IN) for routine testing of patients and employees including both symptomatic testing and surveillance testing. This study includes all samples positive for SARS-CoV-2 RNA by the TaqPath RT-PCR between December 2020 and July 2022, a period that encompasses the SARS-CoV-2 Alpha, Delta, and Omicron variant waves in New York City. This study protocol was reviewed, approved, and the need for informed consent was waived by the MSKCC Institutional Review Board. All methods were carried out in accordance with relevant guidelines and regulations**.**

### SARS-CoV-2 RT-PCR

The TaqPath COVID-19 Combo Kit (Thermo Fisher Scientific) targets the N, S, and ORF genes and was performed on either nasopharyngeal swabs (NPS) per manufacturer’s instructions or on saliva samples as a modified LDT. Samples were reported as positive per manufacturers' instructions which includes detection of at least 2 of the 3 gene targets with a cycle threshold (*C*_t_) value less than 37.

### SARS-CoV-2 whole genome sequencing (WGS)

WGS was performed using the Scripps PrimalSeq-Nextera XT protocol, based on the ARTIC protocol, with some modifications. Sequencing was performed on all available samples with adequate viral load (i.e., *C*_t_ value < 30) or upon special request by the infection control service related to cluster investigations as previously described^10,11^. Briefly, nucleic acids extraction was performed on the KingFisher Flex Magnetic Particle Processor using the MagMAX Viral/Pathogen Nucleic Acid Isolation Kit (Thermo Fisher Scientific, Waltham, MA) with WGS performed as paired end (2 × 150 base pair read) on an Illumina Miseq (Illumina, San Diego, CA USA). Amplicon sequencing was performed using the most current version of the ARTIC primers (version 3 during the Alpha wave and the version 4 during the Omicron wave). The two versions of the ARTIC protocols differ in the sets of primers used and updated versions are used to address possible sequence dropout as the SARS-CoV-2 genome evolves. Primer sets were obtained from Integrated DNA Technologies (Coralville, IA). All whole genome sequences passing quality check were analyzed for lineage assignments using the DRAGEN COVID lineage app based on the Pangolin software (https://github.com/cov-lineages/pangolin; version 4.1.2 pangolin-data 1.13).

### Data availability

All SARS-CoV-2 genomes obtained in this study were uploaded to GISAID (Global initiative on sharing all influenza data) database (https://www.gisaid.org/), EPIS_SET_20220427gr.

### Statistical analysis

Analytical performance including sensitivity, specificity, positive predictive value (PPV), and negative predictive value (NPV) of the SGTF signature for identification of the Alpha and Omicron variants was determined using WGS as the gold standard method. The Cohen’s kappa coefficient (k) was calculated to determine the strength of the agreement between the SGTF signature and WGS and interpreted using the following thresholds: values ≤ 0 as no agreement, 0.01–0.20 as none to minimal agreement, 0.21–0.40 as fair agreement, 0.41–0.60 as moderate agreement, 0.61–0.80 as substantial agreement, and 0.81–1.00 as almost perfect agreement. Sensitivity was defined as the ability of the SGTF signature detection to correctly identify the Alpha and Omicron (B.1.1529, BA.1, BA.4 and BA.5) variants (i.e., true positive) while specificity was defined as the ability of the absence of the SGTF signature to correctly exclude the presence of the Alpha and Omicron variants (i.e., true negative). PPV and NPV were defined as the probability that the presence (PPV) or absence (NPV) of the SGTF signature was associated with the presence or absence of the Alpha or the Omicron (B.1.1529, BA.1, BA.4 and BA.5) variants. PPV and NPV were calculated using both overall prevalence and prevalence during each month of the study period as prevalence of the VOC varied. All analyses were performed using GraphPad Prism version 8.4.2. (GraphPad Software, San Diego, CA).

## Results

**Figure 1:**
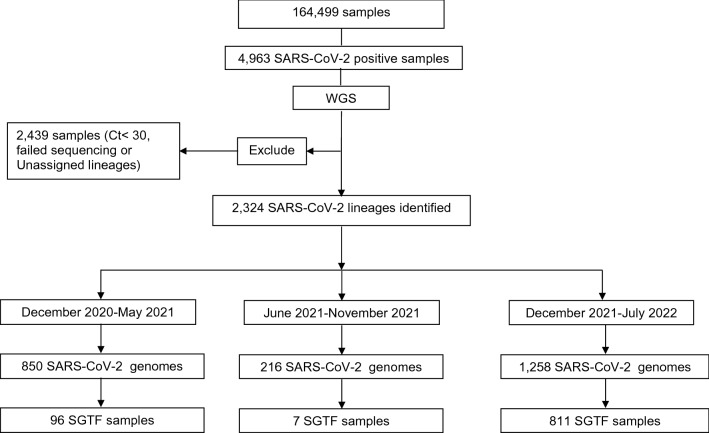
Consort chart showing all samples included in this study and the distribution of samples with whole genome sequences available for each SARS-CoV-2 wave. Alpha wave (December 2020–May 2021), Delta wave (June 2021–November 2021) and Omicron wave (December 2021–July 2022). *WGS* whole genome sequencing. *Ct* RT-PCR cycle threshold, *SGTF* spike gene target failure.

Testing using the TaqPath RT-PCR was performed on 164,499 samples during the study period (Fig. 1). 88,916 samples were tested between December 2020 and May 2021 (Alpha variant wave in NYC); 43,709 samples were tested between December 2021 and July 2022 (Omicron wave: BA.1/BA1.1 December–March 2022; BA.2/BA.2.12.1: March–June 2022; BA.4/BA.5: June–July 2022). Dates between June 2021 and November 2021 corresponded to the Delta variant (B.1.617.2) wave with a total of 31,874 samples tested on TaqPath. The overall SARS-CoV-2 positivity rate on TaqPath was 3.0% (4963/164,499) and 47.5% of samples (2357/4963) were positive for the SGTF signature.

A total of 3056/ SARS-CoV-2 positive samples qualified for NGS testing (i.e., Ct values < 30) and 2324 samples were successfully sequenced and assigned a SARS-CoV-2 lineage (Fig. 1). The overall agreement between the SGTF signature from the TaqPath RT-PCR and NGS for identification of the Alpha and Omicron variants was 99.3% (2307/2324) with a k value of 0.99 suggesting an almost perfect agreement (Table 1). Data for the each of SARS-CoV-2 VOC are summarized in Table 2. During the Alpha wave, 850 SARS-CoV-2 genomes were sequenced including 96 SGTF positive samples with 86/96 (89.5%) classified as Alpha variant (Fig. 1). An additional 7 SGTF positive samples were detected during the Delta wave (7/216 samples) and all 7 were identified as Alpha variant for an overall sensitivity of 100% (95% CI 96.1–100%) and a specificity of 99.6% (95% CI 99.2–99.8%) for the Alpha variant (Table 2). During the Omicron wave (December 2021–July 2022), 1258 SARS-CoV-2 genomes were successfully sequenced including 811 SGTF positive samples with 808/811 (99.6%) classified as Omicron (B.1.1529, BA.1, BA.4 and BA.5) (Fig. 1). Three non-SGTF samples were identified as Omicron (BA.1) by sequencing resulting in an overall sensitivity of 99.6% (98.9–99.9%) and a specificity of 99.8% (95% CI 99.4–99.9%) for Omicron variants (B.1.1529, BA.1, BA.4 and BA.5) (Table 2).

**Table 1:** Performance characteristics of the TaqPath RT-PCR S gene target failure (SGTF) signature for the overall identification of the alpha and omicron variants.

	Next generation sequencing		
	Alpha/omicron VOC	Not alpha/omicron VOC	Total
TaqPath RT-PCR			
SGTF positive	901	13	914
SGTF negative	3	1407	1410
Total	904	1420	2324
Sensitivity% (95%CI)	99.7% (99.0–99.9%)		
Specificity% (95%CI)	99.1% (98.4–99.5%)		
PPV% (95%CI)	98.6% (97.6–99.2%)		
NPV% (95%CI)	99.8% (99.4–99.9%)		
Agreement%, k value	99.3%, 0.98		

*SGTF* spike gene target failure, *N* number of samples in each category, *VOC* variant of concern, *N/A* Not Applicable (i.e., this variant is not (or rarely) expected to be identified using the SGTF pattern). CI: Confidence Interval, k value: Cohen Kappa value. Sensitivity: Percent of samples correctly identified as Alpha VOC or Omicron VOC (B.1.1. 529, BA.1, BA.4, BA.5) based on the presence of the SGTF pattern; Specificity: Percent of sample correctly identified as NOT Alpha VOC or Omicron VOC (B.1.1. 529, BA.1, BA.4, BA.5) based on the absence of the SGTF pattern.

**Table 2:** Performance characteristics of the TaqPath RT- PCR S gene target failure (SGTF) signature for each variant.

SARS-CoV-2 whole genome sequencing	SARS-CoV-2 TaqPath RT-PCR		SGTF performance characteristics	
	SGTF (N)	Not SGTF (N)	Sensitivity% (95%CI)	Specificity% (95%CI)
Alpha VOC ( B.1.1.7 and Q lineages)	93	0	100% (96.1–100%)	99.6% (99.2–99.8%)
Delta VOC ( B.1.617.2 and AY lineages)	0	200	N/A	100% (99.8–100%)
Omicron VOC (B.1.1. 529, BA.1, BA.4, BA.5)	808	3	99.6% (98.9–99.9%)	99.8% (99.4–99.9%)
Omicron VOC ( BA.2, BA.3)	0	361	N/A	100% (99.8–100%)
Other SARS-CoV-2 variants	13	846	N/A	99.4 (98.9–99.7%)
All variants	914	1410	99.7% (99.0–99.9%)	99.0% (98.4–99.5%)

*SGTF* S gene target failure, *N* number of samples in each category, *VOC* variant of concern, *N/A* not applicable (i.e., this variant is not (or rarely) expected to be identified using the SGTF pattern). Sensitivity: Percent of samples correctly identified as Alpha VOC or Omicron VOC (B.1.1. 529, BA.1, BA.4, BA.5) based on the presence of the SGTF pattern; Specificity: Percent of sample correctly identified as NOT Alpha VOC or Omicron VOC (B.1.1. 529, BA.1, BA.4, BA.5) based on the absence of the SGTF pattern.

**Figure 2:**
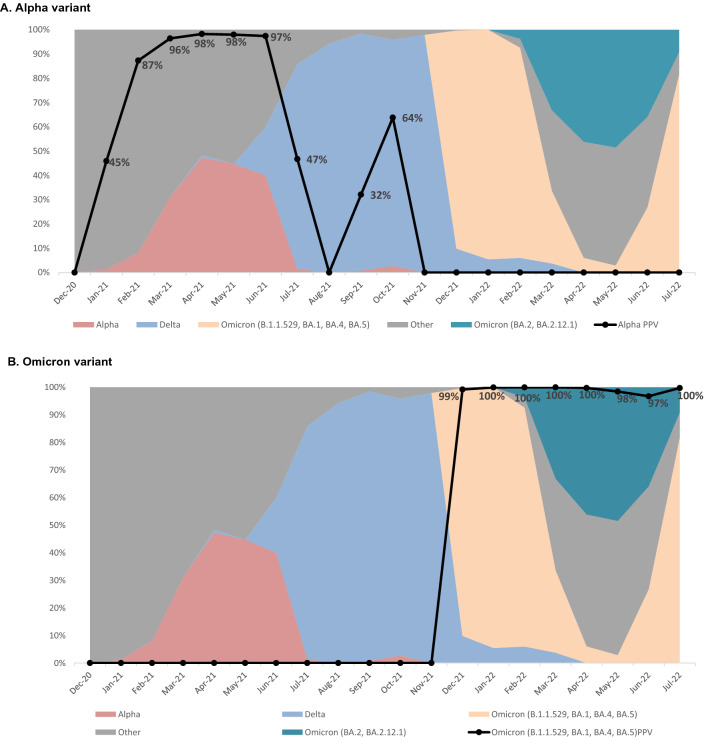
SARS-CoV-2 variants distribution and SGTF positive predictive values. (**A**) Distribution of major variants of concern detected between December 2020 and July 2022 and positive predictive value of the SGTF signature for detection of the Alpha (B.1.1.7) VOC at different time point during the study period. (**B**) Distribution of major variants of concern between December 2020 and July 2022 and positive predictive value of the SGTF signature for detection of the Omicron (B.1.1. 529, BA.1, BA.4, BA.5) VOC at different time point during the study period.

Using a cumulative prevalence for the Alpha variant in New York State of 8%^12^, the PPV and NPV of the SGTF was 83.7% and 100% respectively. For the Omicron variant, with a cumulative prevalence of 54%^13^, the PPV and NPV were 99.8% and 99.5% respectively. Using test positivity rate and all sequenced cases to estimate monthly SARS-CoV-2 variants prevalence at MSKCC, the PPV and NPV of the SGTF signature for the Alpha variant ranged from 45.9 to 100% respectively in January 2021 to 98.3% and 100% in April 2021 (Fig. 2A). For the Omicron variant, with the prevalence rising sharply in December 2021, the PPV ranged from 0% the first week of December to 96.7–99.9% throughout 2022, with a NPV ranging from 96.9 to 99.9% (Fig. 2B).

**Figure 3:**
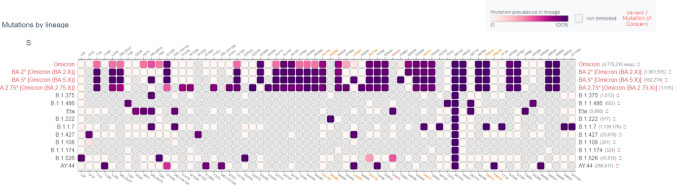
Spike gene mutation prevalence across lineages with SARS-CoV-2 SGTF signature. Global prevalence of SARS-CoV-2 variants detected at MSK with known S gene mutations. Shaded squares reflect the lack of the corresponding mutations in the lineage. The darker the square the highest the prevalence of the particular mutation in the lineage. https://outbreak.info/compare-lineages Figure created 8/17/2022^14–16^.

SARS-CoV-2 variant lineages that were positive for the SGTF signatures but were neither the Alpha nor the Omicron variants are listed in Table 3. For 46% (6/13) of these SGTF positive samples, the S gene del 69–70 that has been associated with the PCR target failure was detected. Two samples, with the B.1.108 lineage, were positive for the S gene del 69–70 although none of the sequences reported to date in GISAID for this variant have been positive for this deletion. For 54% of SGTF positive samples (7/13), no deletions were detected in the S gene but for 43% (3/7), the del 69–70 was previously reported in a small numbers of these lineages (Table 3 and Fig. 3).

**Table 3:** Lineages of SARS-CoV-2 with S gene target failure other than Alpha and Omicrons (N = 13).

Sample	Lineage	S gene amino acid deletion	Global frequency of del69-70 deletion in lineage (%)
EPI_ISL_12203039	B.1.375	S:H69-,S:V70-	95.4
EPI_ISL_12203040	B.1.1.486	None detected	
EPI_ISL_12203041	B.1.1.486	None detected	
EPI_ISL_12203042	B.1.525	S:H69-, S:V70-,S:Y144	93
EPI_ISL_12203043	B.1.222	None detected	
EPI_ISL_5926565	B.1	None detected	2
EPI_ISL_5944669	B.1.108	S:T63-, S:W64-S:F65-S:H66-, S:A67-, S:I68-S:H69-, S:V70-, S:S71-S:G72-, S:T73-, S:N74-S:G75-, S:Y144-	0
EPI_ISL_5944842	B.1.108	S:T63-, S:W64-S:F65-S:H66-, S:A67-, S:I68-S:H69-, S:V70-, S:S71-S:G72-, S:T73-, S:N74-S:G75-, S:Y144-	0
EPI_ISL_2690313	B.1.1.174	None detected	
EPI_ISL_12175123	B.1	None detected	2
EPI_ISL_12150401	B.1.526	S:H69-, S:V70-S:Y144	0.1
EPI_ISL_12150087	AY.44	None detected	0.3
EPI_ISL_12150484	B.1.526	S:H69-, S:V70-	0.1

## Discussion

In this study, we investigated the analytical performance of the TaqPath RT-PCR SGTF signature for preliminary identification of the SARS-CoV-2 Alpha (B.1.1.7) and Omicron variants (B.1.1.529, BA.1, BA.4 and BA.5). The sensitivity and specificity were excellent for both VOC (i.e., greater than 95%) with PPV increasing significantly as the prevalence of each variant increased. Importantly, the NPV was high in our cohort, suggesting that samples in which the S gene target was detected the TaqPath RT-PCR were unlikely to be either Alpha or Omicron (except for the BA.2 and BA.3 subvariant).

Variants specific RT-PCRs, based on the detection of specific single nucleotide polymorphisms (e.g., N501Y) or deletions (e.g., del 69–70), have been published^17–19^. However, with the frequent emergence and decline of SARS-CoV-2 variants during the pandemic, an approach relying on constant update of a variant specific RT-PCR might not be practical for many laboratories and not sustainable for others. Recently referred to as the “S gene advantage”, assays designed to target the S gene have offered a readily available tool to preliminarily infer or rule-out the presence of VOC including Alpha or Omicron. In one report focusing on the Alpha variant, the prevalence of the SGTF signature was shown to be in pace with the emergence of the Alpha variant in Toronto with a specificity of approximately 98% similar to what is reported in the current study^20^. False-positive and false-negative SGTF for the Alpha variant were reported in an early study and attributed to the concomitant high prevalence of another variant with the del 69–70 (B.1.528) and analytical issues with the test (e.g., false amplification signal for the S gene) respectively^21^. We similarly observed a few samples in this study that were false positive and false negative for Alpha and Omicron variants, likely for the same reasons. A recent report from South Africa showed, similar to our study, that the TaqPath SGTF signature had high accuracy to identify Omicron BA.1 sublineage (97.5% confirmed by WGS). In that study, a genotypic assay targeting both the del 69–70 and a K417N was used as the gold standard for identifying Omicron BA.1 and ruling out of the Alpha and Beta variants with a subset of samples confirmed by WGS^22^. Given that the del 69–70 is present in VOC other than Omicron and Alpha, it is important to know the local prevalence of emerging variants for optimal application of the SGTF signature.

Monoclonal antibodies (MAbs) and antiviral drugs are clinically most effective when used early after symptom onset. The neutralizing activity of MAbs depends on the spike mutation profile and is variable for the different VOC, which is the basis for the changing recommendations on Mabs use. For example, In early January 2022, the U.S. FDA revised the emergency use authorization (EUA) for bamlanivimab and etesevimab (Eli Lilly and Company, Indianapolis, IN) and casirivimab and imdevimab (Regeneron Pharmaceuticals Inc., Tarrytown, NY) due to significantly reduced neutralizing activity against the Omicron variant^23^. A blanket mandate (i.e., independent of the SARS-CoV-2 variant identity) to discontinue use was issued given the high prevalence of Omicron, even though some patients with Delta infection during this time may have potentially benefited from this treatment. In this context, the potential use of the SGTF signature to promptly identify patients likely to respond to these treatments or guide preferential use of antiviral drugs over monoclonal antibodies (i.e., SGTF negative patients) might prove useful and overcome some of the uncertainty that has been a barrier to wider Mabs use.

Similarly, early data on Sotrovimab (GlaxoSmithKline and Vir Biotechnology, Inc., Brentford, UK), one of the monoclonal antibody therapies that remains effective against the Omicron variants (BA.1. and BA1.1.), indicates that it may have reduced effectiveness against BA.2^24^. Recent approval of bebtelovimab by the US FDA has provided additional monoclonal antibody treatment with data showing activity against both BA.1. and BA.2. As the prevalence of BA.2 increased, results of the current study showed that the SGTF signature, which is negative for BA.2, would have been valuable in distinguishing between these two Omicron subvariants. The current Omicron wave is driven by the BA.4 and BA.5 subvariants, both of which are positive for the SGTF signature, a characteristic that was useful to monitor, almost in real-time, the change in circulating variants as BA.2 was being replaced in early June 2022 in New York. Thus, simple identification of this genomic signature that provide rapid knowledge of probable circulating variants, has an important practical impact on patient care and contact investigations.

Our study has some limitations. First, it is a single-center study, performed at a tertiary cancer care center and thus our findings may not apply widely to other healthcare centers. However, in addition to our cancer patient population, a significant number of our healthcare workers are tested at our institution and represent a more accurate reflection of SARS-CoV-2 variants circulating in the general population. Second, not every sample was tested using the TaqPath COVID-19 test as the laboratory performed testing using multiple platforms^9–11^; therefore, not all potential SGTF samples were included. Third, most samples sequenced had relatively high viral loads (Ct < 30) and thus data could be biased towards these type of samples.

Despite these limitations, our study reflects one of the largest dataset evaluating the analytical performance of the SGTF signature across two VOCs. Of note, the TaqPath COVID-19 test does not have FDA EUA approval specifically for identification of SARS-CoV-2 VOC to make clinical decision. However, the data presented in this study might be useful to laboratorians, clinicians, and epidemiologists to provide quick, preliminary data on circulating VOC at a local level and thus inform direct patient care.
